# Early Edema Within the Ischemic Core Is Time-Dependent and Associated With Functional Outcomes of Acute Ischemic Stroke Patients

**DOI:** 10.3389/fneur.2022.861289

**Published:** 2022-04-07

**Authors:** Qing Han, Jianhong Yang, Xiang Gao, Jichuan Li, Yuefei Wu, Yao Xu, Qing Shang, Mark W. Parsons, Longting Lin

**Affiliations:** ^1^Department of Neurology, Ningbo First Hospital, Ningbo, China; ^2^Department of Neurosurgery, Ningbo First Hospital, Ningbo, China; ^3^Sydney Brain Center, University of New South Wales, Sydney, NSW, Australia

**Keywords:** edema, net water uptake, multimodal CT, acute ischemic stroke, ischemic core, penumbra, functional outcome

## Abstract

**Objective:**

To investigate the difference in early edema, quantified by net water uptake (NWU) based on computed tomography (CT) between ischemic core and penumbra and to explore predictors of NWU and test its predictive power for clinical outcome.

**Methods:**

Retrospective analysis was conducted on patients admitted to Ningbo First Hospital with anterior circulation stroke and multi-modal CT. In 154 included patients, NWU of the ischemic core and penumbra were calculated and compared by Mann–Whitney *U* test. Correlations between NWU and variables including age, infarct time (time from symptom onset to imaging), volume of ischemic core, collateral status, and National Institutes of Health Stroke Scale (NIHSS) scores were investigated by Spearman's correlation analyses. Clinical outcome was defined using the modified Rankin Scale (mRS) at 90 days. Logistic regression and receiver operating characteristic analyses were performed to test the predictive value of NWU. Summary statistics are presented as median (interquartile range), mean (standard deviation) or estimates (95% confidence interval).

**Results:**

The NWU within the ischemic core [6.1% (2.9–9.2%)] was significantly higher than that of the penumbra [1.8% (−0.8–4.0%)]. The only significant predictor of NWU within the ischemic core was infarct time (*p* = 0.004). The NWU within the ischemic core [odds ratio = 1.23 (1.10–1.39)], the volume of ischemic core [1.04, (1.02–1.06)], age [1.09 (1.01–1.17)], and admission NHISS score [1.05 (1.01–1.09)] were associated with the outcome of patients adjusted for sex and treatment. The predictive power for the outcome of the model was significantly higher when NWU was included (area under the curve 0.875 vs. 0.813, *p* < 0.05 by Delong test).

**Conclusions:**

Early edema quantified by NWU is relatively limited in the ischemic core and develops in a time-dependent manner. NWU estimates within the ischemic core may help to predict clinical outcomes of patients with acute ischemic stroke.

## Introduction

Cerebral edema is defined as a pathological increase in the water mass contained in the brain interstitial space (1). The disruption of water homeostasis can occur in the hyperacute stage after brain ischemia (2) and plays an important role in the pathophysiology of ischemic stroke. Early cerebral edema is a sign of damage to the blood–brain barrier (BBB) and disruption of neuronal ion channels, which contribute to poor clinical outcomes (3–5). However, it is difficult to quantify early cerebral edema after acute stroke.

Computed tomography (CT)-based net water uptake (NWU) is an emerging tool for the quantitative assessment of tissue edema (6, 7). NWU within early infarct was reported as a reliable biomarker identifying patients within the time window of thrombolysis (7, 8) and predicting malignant edema or poor outcome after large vessel occlusion (9–11).

Cerebral edema, quantified by NWU, may provide essential information on the assessment of patients with acute ischemic stroke; however, the relationship between NWU and other important predictors (12–15) such as age, the volume of infarct lesion, National Institutes of Health Stroke Scale (NIHSS) scores, and collateral status need further exploration. In addition, previous studies testing the correlations between NWU and clinical prognosis were limited to patients with large vessel occlusion. It is, therefore, not known whether NWU in the ischemic lesion is predictive of outcome in patients with small-vessel occlusion.

Previous studies have also focused primarily on infarct core (tissue that has already infarcted); however, the occurrence and predictive role of edema in the penumbra (hypoperfused tissue that is at risk of infarction but potentially salvageable) (16) remains unknown.

Therefore, this study was designed to investigate the difference in edema between ischemic core and penumbra, explore the relevant predictors of NWU within early infarct, and test whether it is a reliable predictor of poor outcomes in acute ischemic stroke.

## Methods

### Patients

All patients with acute ischemic stroke who received multi-modal CT imaging on admission between July 2017 and September 2019 at Ningbo First Hospital, China were retrospectively screened for inclusion.

Inclusion criteria for this study were as follows: (1) ischemic core and penumbra in anterior circulation territory confirmed by CT perfusion (CTP) and dynamic CT angiography (CTA), including either large vessel or small vessel occlusion; (2) assessment by NIHSS score on admission; (3) documented infarct time (from symptom onset to admission imaging) except for patients with wake-up strokes. The exclusion criteria included preexisting infarctions, hemorrhage, and any other abnormal alteration of density on admission non-contrast CT (NCCT). Baseline demographic data and clinical characteristics were extracted from the medical records. The modified Rankin Scale (mRS) score after 90 days was extracted from the follow-up database.

The study had institutional ethical approval, and written informed consent was obtained for each patient for their collected data.

### Image Acquisitions

Patients received multi-modal CT on admission, including NCCT, CT angiography (CTA), and CTP on a 320-slice scanner (Toshiba Aquilion ONE, Toshiba Medical Imaging, Tokyo, Japan). The CTP was performed with the following protocol: temporally, 19 time points were obtained, with the acquisition commencing 4 s after non-ionic iodinated contrast injection into an antecubital vein (50 ml, 5 ml/s; Bayer HealthCare, Berlin, Germany). Spatially, 320 axial sections with a thickness of 0.5 mm were obtained, which covered the whole brain (160 mm total coverage). CTP data were processed by using commercial software (MIstar, Apollo Medical Imaging Technology, Melbourne, VIC, Australia). The mathematical model of delay-corrected singular value decomposition was chosen to generate perfusion parameters, including cerebral blood volume (CBV), cerebral blood flow (CBF), mean transit time (MTT), and delay time (DT) (17). The volumes of ischemic lesion, ischemic core, and penumbra were then quantified by appropriate thresholds reported elsewhere (18): DT <3 s for total ischemic lesion, CBF <30% for acute ischemic core, and penumbra measured by the total ischemic lesion volume minus ischemic core volume. CTA data were post-processed on a Vitrea workstation (Vitreafx version 1.0, Vital Images, Minnetonka, MN, United States).

### Image Analysis

**Figure 1:**
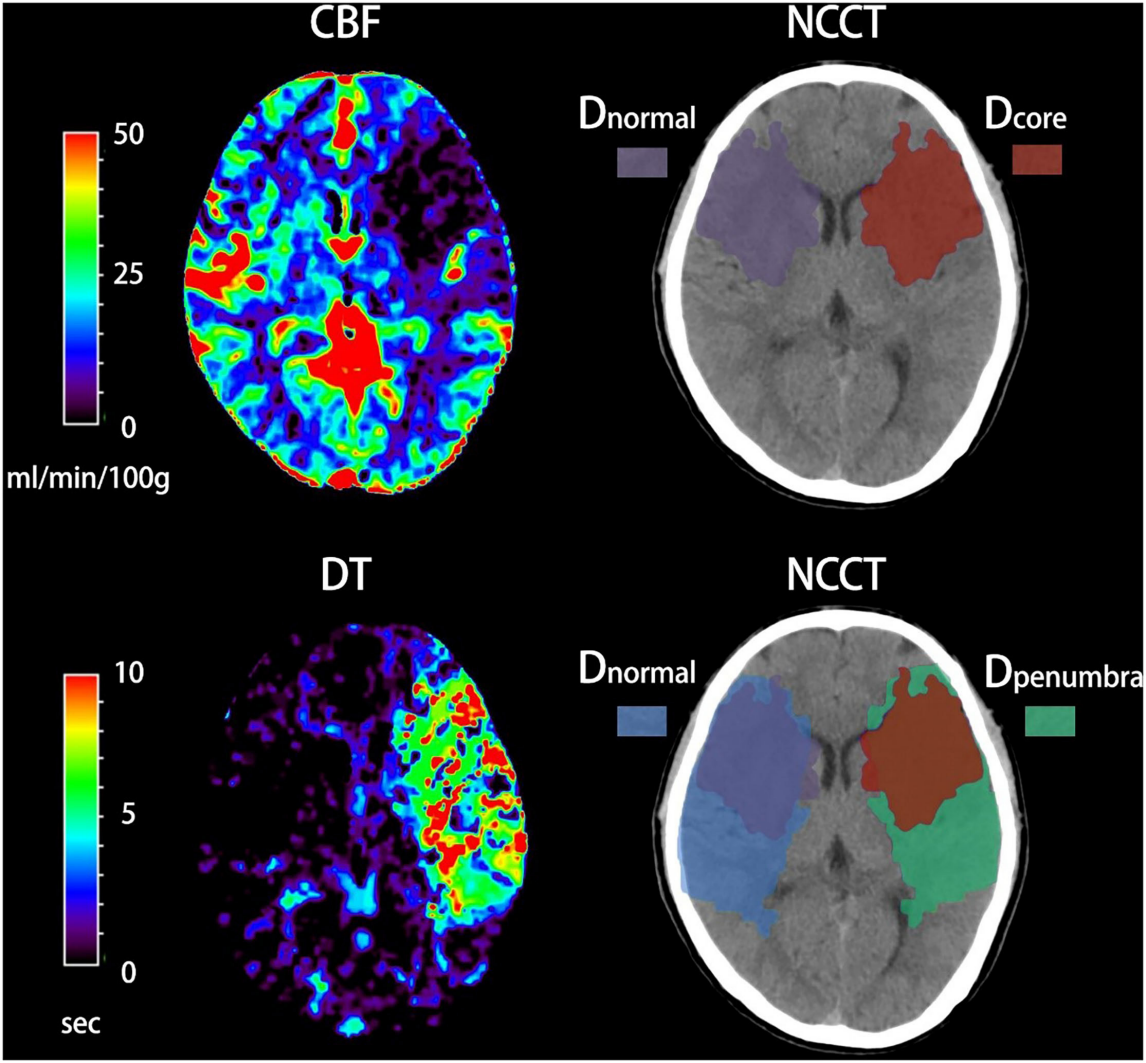
Quantification of net water uptake (NWU) per volume of early infarct in admission non-contrast computed tomography (NCCT). The ischemic core and penumbra were identified by cerebral blood volume (CBV) and delay time (DT) maps based on computed tomography perfusion (CTP). The mean density of early infarct (D_core_ and D_penumbra_) and the normal tissue (D_normal_) derived from a mirrored contralateral region of interest were then calculated.

All imaging measurements were performed using commercial software MIstar (Apollo Medical Imaging Technology, Melbourne, VIC, Australia). NWU was defined as increased volume of water after stroke (Δ*V*_*water*_) per unit volume of the ischemic lesion (*V*_*ischemic*_). A standardized procedure was performed to quantify NWU within early ischemic core based on multi-modal CT. Briefly, regions of interest (ROIs) for ischemic core and penumbra were firstly drawn according to CTP and then automatically mirrored contralateral ROIs were defined as normal tissue. These ROIs were placed on NCCT and were sampled between 20 and 80 Hounsfield units (HU) to exclude voxels including CSF or calcification (Figure 1). All densitometric CT measurements (*D*_*normal*_, *D*_*ischemic*_ including *D*_*core*_ *and D*_*penumbra*_) were then used to calculate NWU according to the following equations (6, 7).


$$\begin{array}{l} NWU\;=\;\frac{\text{Δ}V_{water}}{V_{ischemic}}\;=\;\frac{V_{ischemic}\;-\;V_{normal}}{V_{ischemic}}\;\\ \;=\;\left(\text{1 − }\frac{D_{ischemic}}{D_{normal}}\right)\;\times \text{ 100}\% \end{array}$$

### Statistical Analysis

According to the results of Kolmogorov–Smirnov test, all numerical variables were described as mean [standard deviation (SD)], or median [interquartile range (IQR)]. Categorical data were expressed as numbers (percentages). The NWU within ischemic core and penumbra were calculated and then compared by Student's *t*-test or Mann–Whitney *U* test as appropriate. The relationship between NWU within the ischemic core and other baseline variables including infarct time (time from symptom onset to imaging), age, NNHISS score, the volume of ischemic core, and collateral status were assessed using Pearson's correlation analysis or Spearman's correlation analysis as appropriate. Collateral status was classified by a modified version of the ASITN/SIR (American Society of Interventional and Therapeutic Neuroradiology/Society of Interventional Radiology) collateral scale on dynamic CTA (19, 20), and it was further dichotomized into good collateral (ASITN/SIR score 3–4) and poor collateral (ASITN/SIR score 0–2) status. The relationship between NWU within the ischemic core and collateral status (good vs. poor collateral) was further tested on Mann–Whitney *U* test.

We defined poor functional outcome (functional dependence or death) as a modified Rankin Scale (mRS) of ≥3 at 90 days. Baseline data were compared after dichotomization into mRS score 0–2 and mRS score 3–6 using the Mann–Whitney U Test for metric variables and Chi-square test for categorical variables. To investigate the predictive value of ischemic core NWU and other variables for the likelihood of the poor functional outcome, we performed binary logistic regression analyses presenting odds ratio (OR) estimates along with 95% confidence intervals (CI). The diagnostic power of variables and predictive models were assessed by univariate receiver operating characteristic (ROC) curve analysis. The ROC comparisons between different predicting models were performed using the DeLong test.

A two-tailed *p* < 0.05 was considered statistically significant. All Statistical analyses were performed using SPSS Statistics for Windows, Version 22.0 (IBM Corp., Armonk, NY, USA) and MedCalc (version 9.2.1; Mariakerke, Belgium).

## Results

**Figure 2:**
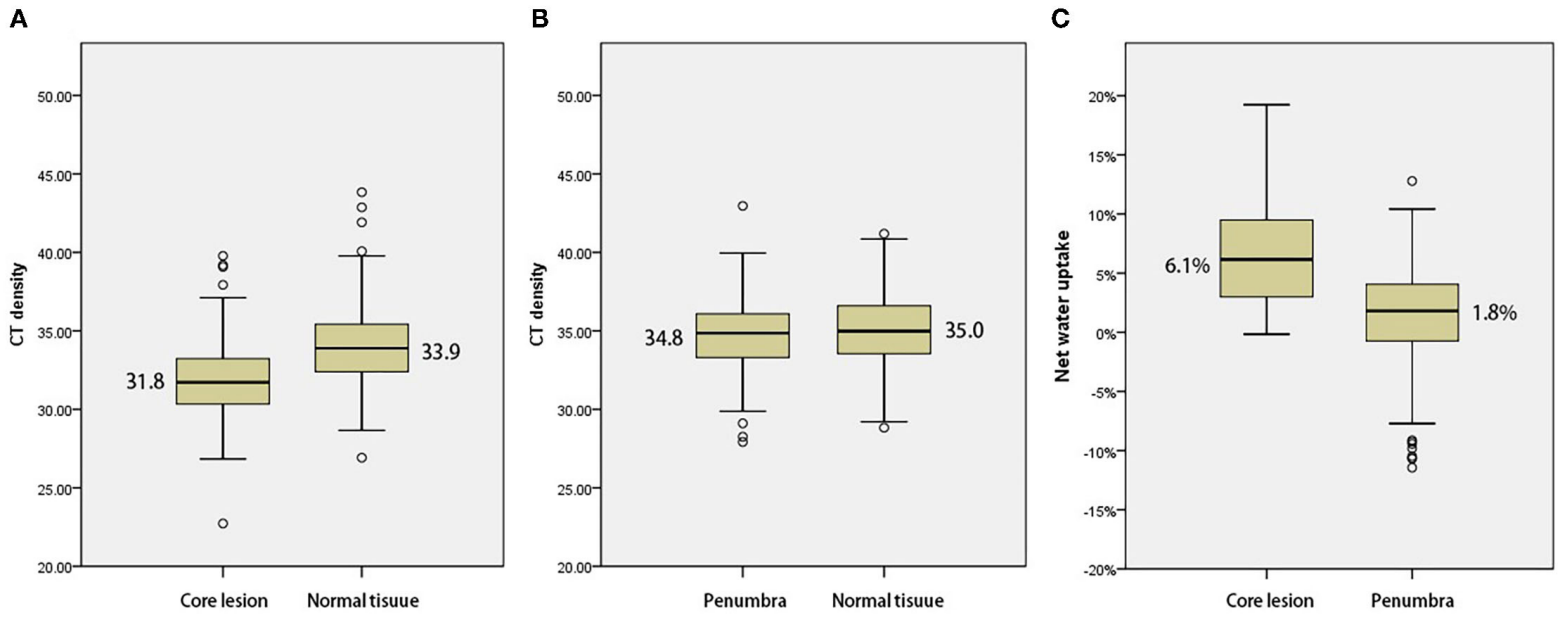
**(A)** Comparison with mean density of the ischemic core and normal tissue. **(B)** Comparison with mean density of penumbra and normal tissue. **(C)** Comparison of net water uptake (NWU) with ischemic core and penumbra.

Of the 248 screened patients, 154 fulfilled the inclusion criteria of which 127 had documented the time of symptom onset and 27 had wake-up strokes. In all 154 included patients, NWU within the ischemic core, penumbra, and contralateral healthy tissue were calculated and then compared using the Mann–Whitney U test. The density of ischemic core on admission NCCT was significantly lower than that of contralateral normal tissue [median 31.8 HU (30.3–33.2) vs. median 33.9 HU (32.4–35.4), *p* < 0.001, Figure 2A). The density of penumbra was also lower than that of contralateral normal tissue but this difference was not statistically significant [median 34.8 HU (33.3–36.1) vs. median 35.0 HU (33.5–36.6), *p* = 0.158, Figure 2B]. The NWU within the ischemic core was significantly higher than that of the penumbra [median 6.1% (2.9–9.2%) vs. 1.8% (−0.8 to 4.0%), *p* < 0.001, Table 1 and Figure 2C].

**Table 1:** Net water uptake in different categories of lesion, infarct time, core volume, and collateral status.

**Category**	**Net water uptake % median (IQR)**
**Region**	
Ischemic core	6.1 (2.9–9.2)
Penumbra	1.8 (−0.8–4.0)
**Time from onset to CT**	
0–3 h, *n* = 32	3.4 (0.9–7.3)
3–6 h, *n* = 74	6.2 (3.3–9.0)
6–24 h, *n* = 21	9.2 (4.2–13.3)
**Volume of ischemic core**	
0–30 ml, *n* = 76	4.5 (1.9–7.6)
30–50 ml, *n* = 35	7.5 (3.6–11.4)
50–70 ml, *n* = 18	5.9 (2.0–7.9)
>70 ml, *n* = 25	6.8 (4.0–9.3)
**Collateral status**	
ASITN/SIR 0–2, *n* = 105	6.4 (3.2–9.3)
ASITN/SIR 3–4, *n* = 49	4.4 (1.8–9.2)

*IQR, interquartile range; CT, computed tomography; ASITN/SIR, modified version of American Society of Interventional and Therapeutic Neuroradiology/Society of Interventional Radiology collateral scale on dynamic CT angiography*.

**Figure 3:**
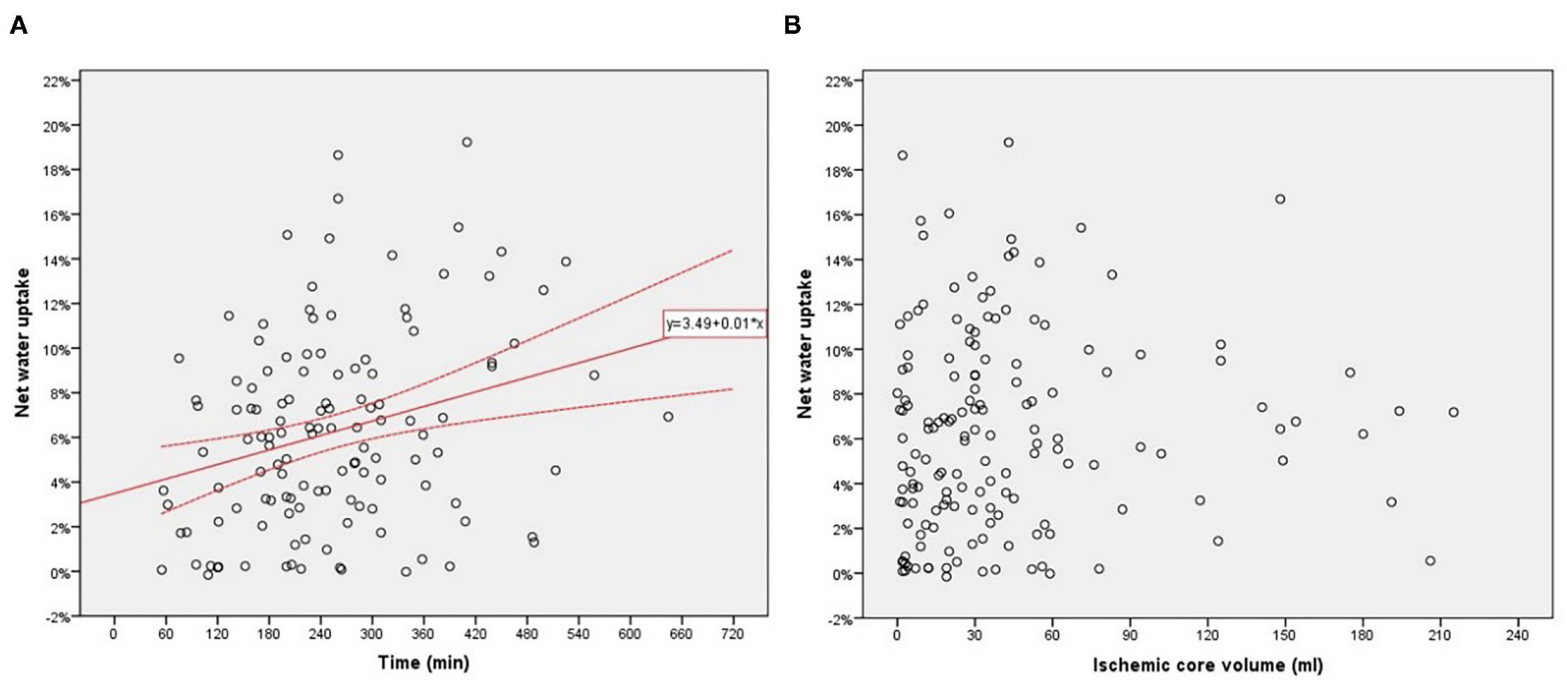
**(A)** Relationship between net water uptake (NWU) within the ischemic core and infarct time from onset to imaging. **(B)** Relationship between NWU within ischemic core and core volume.

A Spearman test was performed to investigate correlations between NWU within ischemic core and age, admission NHISS score, and volume of ischemic core in 154 patients. The correlation between NWU and infarct time was analyzed in those 127 patients with a documented time of onset. There was a significant correlation between NWU and infarct time (*p* = 0.004, Figure 3A). However, the NWU within the ischemic core was not correlated with age (*p* = 0.954), admission NHISS score (*p* = 0.821), ASITN/SIR score (*p* = 0.287), the volume of the ischemic core (*p* = 0.094, Figure 3B). There was no significant difference of NWU between the good collateral group (ASITN/SIR score 3–4) and poor collateral group (ASITN/SIR score 0–2) on Mann–Whitney *U* test (*p* = 0.137), although patients with good collateral showed a tendency of lower NWU (4.4 vs. 6.4, Supplementary Figure 1). The value of NWU in different categories of infarct time, core volume, and collateral status are summarized in Table 1.

Of the 154 patients, 128 were followed up at 90 days; these 128 patients were divided into good outcome (mRS score 0–2, *n* = 53) and poor outcome groups (mRS score 3–6, *n* = 75). The main characteristics of the two groups are summarized in Table 2. The NWU within the ischemic core in patients with good outcomes was significantly lower compared to patients with poor outcomes. Patients with the good outcome also tended to be younger, have a smaller volume of the ischemic core and lower NIHSS score at baseline, and were less likely to be female than those with poor outcome. The infarct time and treatment of the two groups did not significantly differ.

**Table 2:** Characteristics of patients with anterior circulation infarct stratified by prognosis.

**Characteristics**	**Good prognosis (mRS at 90 days 0–2)**	**Poor prognosis (mRS at 90 days 3–6)**	***p*-value^a^**
Subjects, *n* (%)	53 (41.4)	75 (58.6)	
Female sex, *n* (%)	18 (34.0)	40 (53.3)	0.033^*^
Age in yeas, median (IQR)	68 (56–76)	74 (66–81)	0.009^*^
Admission NHISS, median (IQR)	15 (9–18)	20 (14–25)	<0.001^*^
Time from onset to CT, median h (IQR)^b^	4.2 (2.8–4.9), n = 45	3.8 (2.9–5.7), n = 62	0.464
Volume of ischemic core, median ml (IQR)	12 (4–30)	42 (22–62)	<0.001^*^
Treatment			0.162
Mechanical thrombectomy, *n* (%)	19 (35.8)	40 (53.3)	
DPT, median min (IQR)	93 (80–127)	97 (81–132)	
Intravenous lysis, *n* (%)	18 (34.0)	22 (29.3)	
DNT, median min (IQR)	49 (36–61)	52 (32–64)	
Both, *n* (%)	12 (22.6)	8 (10.7)	
None, *n* (%)	4 (7.5)	5 (6.7)	
Net water uptake, median % (IQR)	4.1 (1.2–7.4)	7.2 (4.4–10.9)	<0.001^*^
Net water uptake/time, median %/h (IQR)^b^	1.1 (0.3–2.1), *n* = 45	1.5 (1.0–2.2), *n* = 62	0.059

a *Difference between good and poor prognosis groups*.

b *Twenty-one patients with wake-up strokes were excluded due to unknown time from onset*.

* *Statistical difference between two groups*.

*mRS, modified Rankin scale; CT, computed tomography; IQR, interquartile range; NIHSS, National Institutes of Health Stroke Scale; DPT, door-to-puncture time; DNT, door-to-needle time*.

We performed multivariate logistic regression analysis to determine the effects of NWU within the ischemic core, age, sex, ischemic core volume, admission NHISS score, and treatment on likelihood of poor functional outcome. The result showed the NWU within ischemic core [odds ratio = 1.23 (95% CI 1.10–1.39)], volume of ischemic core [odds ratio = 1.04 (95% CI 1.02–1.06)], age [odds ratio = 1.09 (95% CI 1.01–1.17)] and admission NHISS score [odds ratio = 1.05 [95% CI 1.01, 1.09)] were independently and significantly associated with poor functional outcome while sex and treatment were not (Table 3).

**Table 3:** Binary logistic regression and ROC curve analysis to probability of prognosis after anterior circulation infarct.

	**OR (95%CI)**	***P*** **-value^a^**	**AUC (SE)**	**Sensitivity**	**Specifity**
**Variable^b^**
Net water uptake	1.23 (1.10–1.39)	<0.001	0.681 (0.047)	69.3%	60.4%
Ischemic core volume	1.04 (1.02–1.06)	<0.001	0.778 (0.041)	61.3%	83.0%
Admission NHISS	1.09 (1.01–1.17)	0.024	0.716 (0.046)	57.3%	77.4%
Age	1.05 (1.01–1.09)	0.021	0.636 (0.049)	66.7%	56.6%
**Combined model**					
Net water uptake, Ischemic core volume, Admission NHISS, Age			0.875 (0.031)	74.7%	88.7%
Ischemic core volume, Admission NHISS, Age			0.813 (0.038)	73.3%	79.2%

a *Significance of association with poor prognosis (defined as a score on the modified Rankin scale at 90 days of 3–6) by binary logistic regression*.

b *The gender and treatment included in binary logistic regression were not significant so not listed in the table*.

*ROC, Receiver operating characteristic; OR, Odds ratio; AUC, Area under curve; SE, Standard error; NIHSS, National Institutes of Health Stroke Scale*.

**Figure 4:**
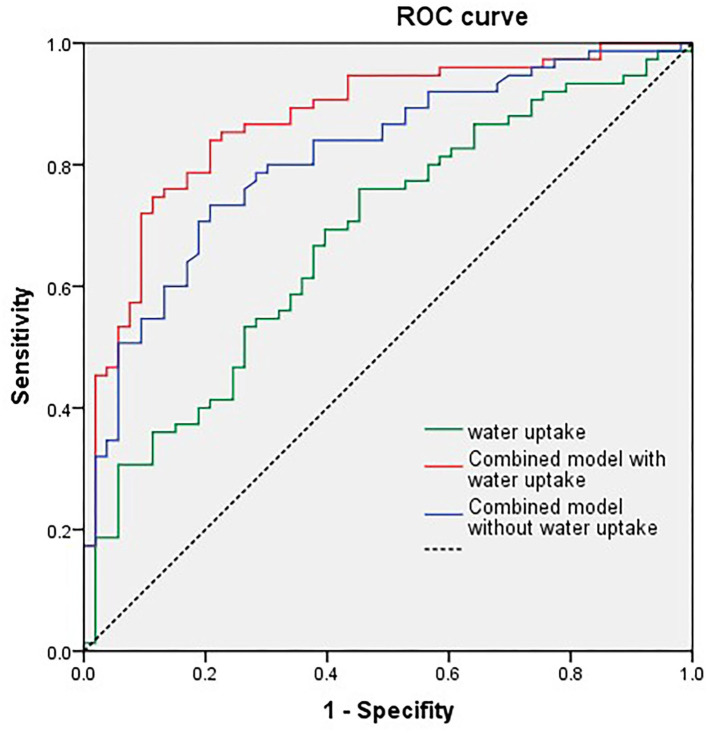
Univariate receiver operating characteristic (ROC) curve analysis of single net water uptake (NWU), combined model with NWU, National Institutes of Health Stroke Scale (NIHSS) score, ischemic core volume, and age.

We tested the power of NWU within the ischemic core to predict the poor functional outcome by the area under the curve (AUC) after receiver operating characteristic curve (ROC) analysis. The AUC of the model including NWU (NWU within ischemic core, volume of ischemic core, age, and NHISS score) was 0.875 while the AUC of the model without NWU was 0.813 (Table 3 and Figure 4). The power of the model including NWU to predict poor functional outcomes was significantly higher than that of the model without NWU according to the DeLong test (*p* = 0.021).

## Discussion

This study confirmed an important role for acute lesion edema, quantified by NWU, in predicting stroke outcomes. One of the main findings of this study was that edema was affected by the severity of ischemia, with limited NWU detected in the ischemic penumbra but significantly higher NWU detected in the ischemic core. Moreover, the severity of edema was not predicted by core volume, but rather by infarct time. This study indicates that the more late a patient with stroke is presented, the more severe is the edema within the ischemic core.

A novel finding of this study was the lack of significant edema in the penumbra region. Previous studies measured NWU either in the whole hypoperfused region (combining both core and penumbra into a single measurement) or ischemic core only. The lack of edema detected in the penumbra may relate to the slower progression of pathophysiological pathways compared to the core. Processes such as ATP depletion, reactive oxygen species generation, oxidative membrane injury, ionic imbalance, and tissue edema develop in the penumbra over several hours or days (21, 22). Previous ultrastructural studies also demonstrated that the degree of edema, degeneration of neurons, glia, and capillaries decreased gradually from ischemic core to penumbra (23, 24). Although this study confirmed previous findings of significantly increased NWU within the ischemic core, the NWU detected was lower compared to previous studies (7, 9, 25, 26). This is probably a result of different thresholds of ischemic core on CTP measurements. Most previous studies used a more severe threshold of CBV ≤6/100 ml while we defined ischemic core using CBF <30%. As NWU gradually decreased based on the degree of perfusion, we can also assume that the early tissue edema would depend on the severity of ischemia within the infarct core.

This study emphasizes the importance of treating stroke patients early. When treated early, further development of edema in the ischemic tissue could be prevented. Another novelty of this study was the discovery of the positive relationship between early edema and infarct time. With prolonged time to treatment, early edema developed, as demonstrated by increased NWU value within the ischemic core. Infarction and edema are dynamic processes with time-dependent development, and this study demonstrated that the severity of edema increased with increased time from onset to reperfusion. It was demonstrated that early edema within the ischemic core could help predict the functional outcome of patients. The high value of NWU within the ischemic core indicated the severity of ionic edema and vasogenic edema, which in turn is related to early damage to the BBB, ion channel disturbance, and other complex mechanisms (3–5), which all may contribute to the poor outcome of patients. All of these findings emphasize the importance of early treatment inpatients with acute stroke.

Besides the infarct time, other factors may relate to early edema. The collateral status reflects the cerebral microperfusion status of patients with acute ischemic stroke, which is linked to edema formation and core growth (27). A previous study showed an association between favorable venous flow profiles and ischemic lesion NWU growth (28). Favorable tissue-level collaterals were also reported predicting less ischemic lesion NWU after performing thrombectomy in patients with large vessel occlusion (29). We did not find a statistically significant relation between modified ASITN/SIR score on dynamic CT and NWU, but there was the tendency of different NWU between good collateral and poor collateral groups. More included cases and appropriate assessments on tissue-level collaterals together with venous outflow (27) may strongly approve the relation between NWU and collateral status of patients with ischemic stroke. Another factor is ischemic core volume, which is recognized as the most essential variable influencing prognosis. In the current study, no significant relation was found between core volume and NWU. This might indicate that NWU within the ischemic core, besides core volume, is a relatively independent factor associated with the functional outcome of patients with ischemic stroke. Finally, factors not assessed in the study, such as admission blood glucose (30, 31), may also affect early edema of ischemic tissues.

The clinical applications of this study include the following two aspects. First, measuring NWU in addition to ischemic core volume for acute stroke management was valuable. Ischemic core volume measured in CTP is not the only imaging biomarker predicting clinical outcomes. Ischemic core volume combined with NWU provides better prognostic value after acute stroke. In addition to admission of NWU, quantitative measures of NWU 24–48 h after treatment and NWU growth (difference between NWU after treatment and on admission) seem to have a significant impact on clinical outcomes as well (29). Secondly, it is important to combine NCCT and CTP when evaluating patients with hyperacute stroke. NCCT has additional value as our study has indicated early edema, quantified by NWU on NCCT, is a potential biomarker to improve the delineation between ischemic core and penumbra.

Our study has several limitations. First, we retrospectively screened patients from a single center. Not every patient with acute ischemic stroke received multi-modal CT imaging. Moreover, patients with mild symptoms and low admission NHISS scores intended to refuse CTP and were therefore not included in the study. This bias may influence the reliability or generalizability of our findings. Secondly, some potential variables related to prognosis after stroke such as hyperglycemia (30, 31) were not included in our logistic regression analyses due to the lack of admission data. Prospective validation is needed to confirm the predictive power of NWU for functional outcomes. Thirdly, CT density measurements of normal tissue relied on automatically mirrored contralateral ROI. This process might result in inaccuracies as the NCCT images were not absolutely symmetrical. However, this method of CT density measurements had been proved reliable previously (6). Finally, the estimates based on the CTP map in this study might be distinct from studies using other CTP processing software packages.

## Conclusion

The early edema after acute stroke quantified by NWU was relatively limited in the ischemic core region anddevelop in a time-dependent manner. The quantified edema within the ischemic core could help predict functional outcomes. These findings emphasize the importance of timely and early treatment of patients with stroke, thereby indicating that early edema quantification using ischemic lesion NWU may be a valuable imaging biomarker based on multimodal CT.

## Data Availability Statement

The raw data supporting the conclusions of this article will be made available by the authors, without undue reservation.

## Ethics Statement

The studies involving human participants were reviewed and approved by the Ethics Committee of Ningbo First Hospital. The patients/participants provided their written informed consent to participate in this study.

## Author Contributions

QH and JY: research project conception, study design, organization, execution, statistical analysis, and writing of the manuscript draft. XG: organization, execution, and study design. JL, YW, YX, and QS: patients' enrolment and follow up and acquisition of data. LL and MP: research project conception, study design, statistical analysis, review and critique, and manuscript revision of the draft.

## Funding

This work was supported by the Medicine and Health Science and Technology Projects of Zhejiang Province (2022KY1098), Clinical Research Fund Project of Zhejiang Medical Association (2021ZYC-A10), and Ningbo Health Branding Subject Fund (PPXK2018-04).

## Conflict of Interest

The authors declare that the research was conducted in the absence of any commercial or financial relationships that could be construed as a potential conflict of interest.

## Publisher's Note

All claims expressed in this article are solely those of the authors and do not necessarily represent those of their affiliated organizations, or those of the publisher, the editors and the reviewers. Any product that may be evaluated in this article, or claim that may be made by its manufacturer, is not guaranteed or endorsed by the publisher.

## Supplementary Material

Supplementary Figure 1 Comparison of net water uptake (NWU) with poor collateral and good collateral patients.
