# Supplementary figures and images for: Early Edema Within the Ischemic Core Is Time-Dependent and Associated With Functional Outcomes of Acute Ischemic Stroke Patients
Source: Front Neurol. 2022 Apr 7;13:861289. doi: 10.3389/fneur.2022.861289 (PMC9021998; doi:10.3389/fneur.2022.861289)

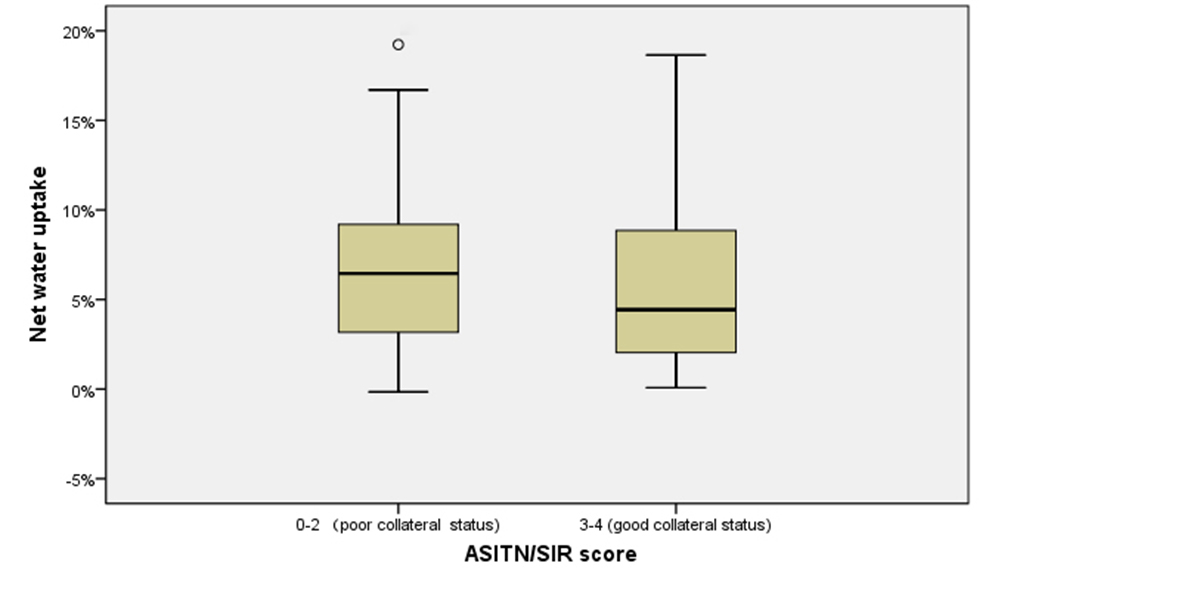

Supplement: Supplementary Figure 1 — Comparison of net water uptake (NWU) with poor collateral and good collateral patients. [file Image_1.JPEG]
